# Fe-HBED Analogs: A Promising Class of Iron-Chelate Contrast Agents for Magnetic Resonance Imaging

**DOI:** 10.1155/2019/8356931

**Published:** 2019-12-20

**Authors:** Brian C. Bales, Brian Grimmond, Bruce F. Johnson, M. Todd Luttrell, Dan E. Meyer, Tatyana Polyanskaya, Michael J. Rishel, Jeannette Roberts

**Affiliations:** GE Research, Niskayuna, NY 12309, USA

## Abstract

Contrast-enhanced magnetic resonance imaging is an essential tool for disease diagnosis and management; all marketed clinical magnetic resonance imaging (MRI) contrast agents (CAs) are gadolinium (Gd) chelates and most are extracellular fluid (ECF) agents. After intravenous injection, these agents rapidly distribute to the extracellular space and are also characterized by low serum protein binding and predominant renal clearance. Gd is an abiotic element with no biological recycling processes; low levels of Gd have been detected in the central nervous system and bone long after administration. These observations have prompted interest in the development of new MRI contrast agents based on biotic elements such as iron (Fe); Fe-HBED (HBED = *N,N*′-bis(2-hydroxyphenyl)ethylenediamine-*N,N*′-diacetic acid), a coordinatively saturated iron chelate, is an attractive MRI CA platform suitable for modification to adjust relaxivity and biodistribution. Compared to the parent Fe-HBED, the Fe-HBED analogs reported here have lower serum protein binding and higher relaxivity as well as lower relative liver enhancement in mice, comparable to that of a representative gadolinium-based contrast agent (GBCA). Fe-HBED analogs are therefore a promising class of non-Gd ECF MRI CA.

## 1. Introduction

Contrast-enhanced magnetic resonance imaging is an “essential tool for disease diagnosis and management” [1]. Currently, marketed clinical magnetic resonance contrast agents are Gd chelates. It has recently been observed in humans and preclinical models that low levels of Gd can be detected in tissue, particularly in the central nervous system following administration of multiple doses of gadolinium-based contrast agents (GBCA) [2–4]. While adverse health outcomes have not been associated with this retention, the observation has revived interest in MRI active chelates of biotic metals; candidates include manganese [5, 6] and iron [7–10]. Manganese [11] and iron [12] are essential to living organisms, which have consequently developed dedicated biological pathways for achieving homeostasis. Iron is of particular interest because iron metabolism [12, 13] is well understood. In addition, intravenous administration of the chelate iron gluconate (125 mg or 250 mg Fe) is a well-tolerated intervention for anemia [14, 15]. In this chelate, Fe is weakly held by gluconate, consistent with the intention to deliver iron in a usable form to relieve anemia; this reduces concerns associated with any escape of low levels of free Fe from a rapidly excreted iron-based contrast agent (IBCA) in which the Fe is tightly chelated.

There are two key challenges for IBCA, both of which can be addressed via appropriate design of the chelate structure. Iron chelates typically provide less signal than gadolinium chelates on a per metal atom basis [7, 8, 16]. Lower signal per chelate molecule can be addressed by dosing more iron chelate. However, signal can be strongly influenced by chelate structure [7, 17], and so the performance of iron chelates may be improved by modifying the chelate structure to improve their relaxivity properties. It should also be noted that the contrast advantage of gadolinium over iron chelates may be partially mitigated at the higher field strengths that are more commonly used today than when the first MRI CAs were developed and commercialized; indeed, that is one reason why Gd was selected over Mn or Fe some 30 years ago [7, 8].

While iron is an endogenous element, iron chelates can nonetheless be toxic at sufficiently high doses; one form of toxicity is derived from the ability of the iron chelate to catalyze oxidative Fenton chemistry to produce hydroxyl radicals which are deleterious to biomolecules. The structure of the chelate strongly affects the ability of an iron chelate to undergo redox chemistry and catalyze Fenton chemistry [18, 19]. Iron chelates are known that do not appreciably catalyze Fenton chemistry and, therefore, minimize an important route by which iron complexes can be toxic.

The primary mechanism by which GBCA provides *T*_1_-weighted MRI signal requires binding of water to the metal ion (the inner sphere). Unfortunately, iron, in a coordinatively saturated iron chelate that does not catalyze Fenton chemistry, does not have a site available for binding to water. Such a chelate relies upon interaction of water with the ligand that binds the iron (the second sphere) to generate signal; this interaction is influenced by a ligand structure; modification of the ligand to increase interaction with water provides a route to improve relaxivity [20].

Fe-HBED is a promising parent chelate for a IBCA; HBED binds Fe[III] tightly, with a log *K* of 39.7 [21]. It is a coordinatively saturated complex that inhibits Fe-catalyzed radical generation in vitro [22–24]. The ligand, HBED, has been evaluated and found effective and well tolerated in preclinical and clinical studies as chelation therapy for iron overload [25–27].

Fe-HBED binds appreciably to human serum albumin and has been evaluated as a liver-specific MRI CA in preclinical studies [28–30]. While this is of clinical utility for liver-specific imaging needs, the most widely used MRI CAs are ECF agents (as opposed to mixed ECF-liver or liver-specific agents). Clinically approved ECF agents are hydrophilic Gd chelates with limited binding to serum proteins; they permeate from vasculature to distribute rapidly to interstitial space and are primarily eliminated via the kidneys. Given the desire for an alternative to GBCA and the promise of Fe-HBED, modification of Fe-HBED analogs for improved imaging performance has been reported [10].

The goal of the work reported here was to modify HBED to make an ECF IBCA with improved contrast and lower serum protein binding relative to Fe-HBED, while maintaining key benefits of HBED—namely, tight Fe binding and an iron chelate inert as an oxidative catalyst. The approach taken was to add hydroxyl groups and/or charge to the ligand to increase hydrophilicity of Fe-HBED analogs and thereby reduce serum protein binding. It was hypothesized that increasing ligand hydrophilicity would also increase *r*_1_ because relaxation is affected by the number of water molecules interacting with the HBED ligand, their distance from the chelated iron, and their residence time [31].

## 2. Materials and Methods

### 2.1. Chemical Synthesis

**Figure 1:**
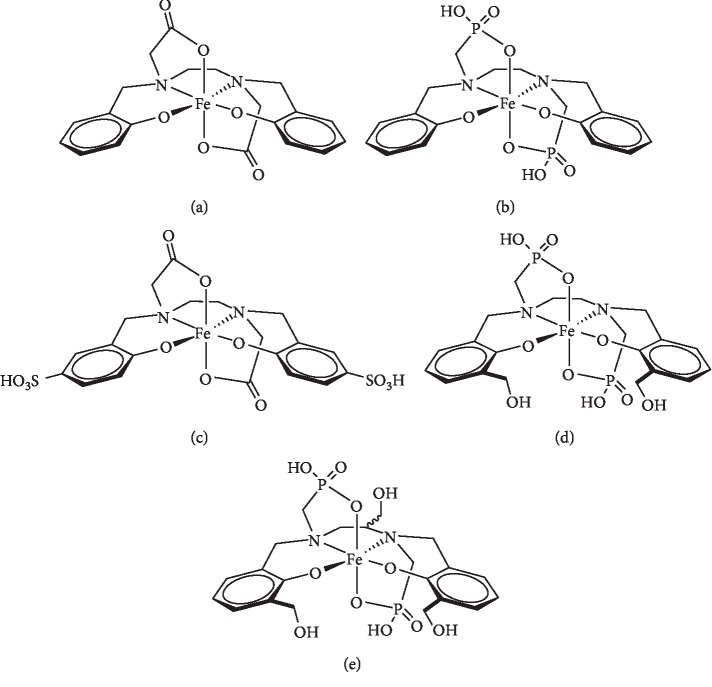
Fe chelates evaluated as ECF MR CA: (a) Fe-HBED (**Fe-1**); (b) Fe-HBEDP (**Fe-3**); (c) Fe-SHBED (**Fe-2**); (d) Fe-HBEDP-(CH_2_OH)_2_ (**Fe-4**); (e) Fe-HBEDP-(CH_2_OH)_3_ (**Fe-5**).

Procedures for the preparation of compounds listed in Figure 1 are shown in the supplemental material ([Supplementary-material supplementary-material-1]).

### 2.2. Analytical Characterization

#### 2.2.1. Relaxivity Determination

A stock solution having a concentration of 1 mM of the CA of interest was prepared in phosphate-buffered saline (PBS), and the iron concentration was verified by elemental analysis. Separate 0.75 mM, 0.50 mM, and 0.25 mM samples were prepared from the stock by dilution in PBS, and *T*_1_ and *T*_2_ relaxations times were recorded in triplicate for each sample on a Bruker minispec mq60 instrument (60 MHz, 40°C). The relaxivities (*r*_1_ and *r*_2_) were obtained as the gradient of 1/*T*_*x*_ (*x* = 1, 2) plotted against Fe chelate concentration following linear least squares regression analysis.

#### 2.2.2. Ascorbic Acid Oxidation Assay

The UV-Vis spectrum of an ascorbic acid solution (67 *μ*M and 12 *μ*g/mL) in PBS (3 mL) was recorded. The absorbance intensity at *λ*_max_ = 265 nm was observed. An aliquot (30 *μ*L) of the iron chelate of ethylenediaminetetraacetic acid (Fe-EDTA) in PBS (2 mM, 0.7 mg/mL) was added to afford a catalytic quantity of Fe-EDTA (20 *μ*M, 30 mol%) with respect to ascorbic acid. The absorbance intensity (*λ*_max_ = 265 nm) was recorded at intervals of 1 minute for a period of 45 minutes and the data normalized to the *t*_0_ absorbance. The experiment was then repeated identically using a solution of the iron chelate complex under investigation (20 *μ*M, 30 mol%), and the results were plotted as a function of the percent initial ascorbic acid signal versus elapsed time (min).

#### 2.2.3. Serum Protein Binding Assay

A 5 mM stock solution of the iron chelate complex under investigation was prepared in PBS, and the UV/visible spectrum of the solution was acquired to determine the wavelength of the absorbance maximum (*λ*_max_). An aliquot of the stock solution (500 *μ*L) was added to a PBS solution (2 mL) containing bovine serum albumin (8 wt.%). A control sample was prepared by diluting a second aliquot of the stock solution (500 *μ*L) with PBS (2 mL). The samples were vortexed briefly and then allowed to stand for 1 hour at room temperature. At the stipulated time, the resulting solutions were transferred to Amicon Ultra filters (4 mL, MWCO = 30 kDa). The solutions were centrifuged (3000 rcf, 15 min), and the permeate was taken directly for UV-Vis measurement. The wavelength and intensity of *λ*_max_ in the visible region for the solution were recorded. The relative amount of free and protein-bound agent was calculated from the *λ*_max_ intensity ratio of the assay to control samples.

### 2.3. Imaging, Biology, and Data Analysis

#### 2.3.1. Imaging Studies

All animal experiments were conducted in accordance with policies of the GE Research Institutional Animal Care and Use Committee under an approved Animal Care and Use Protocol.

Mice used for naïve imaging were 6-week-old female Swiss Webster mice (SW-F) purchased from Taconic Biosciences (Rensselaer, NY). Mice used for the tumor imaging studies were 6-week-old female CD1 nude mice (Crl:CD1-*Foxn1*^*nu*^) purchased from Charles River Laboratories (Wilmington, MA). Cohort number for evaluation of each iron chelate CA was *n* ≥ 5 for all groups except Fe-HBED, which was *n* = 3.

In preparation for tumor-imaging studies, animals were subcutaneously injected with 2 × 10^6^ C6 glioma cells in Matrigel® to their left flanks. Tumors were monitored daily, and the mice were imaged when the tumors reached a diameter of approximately 0.5–1 cm in their longest axis. The C6 glioma cell line (Catalogue #CCL-107) was purchased from ATCC (Manassa, VA). Cells were cultured in F-12K media with 15% horse serum, 25% FBS, and 1% penicillin-streptomycin.

For imaging of both naïve and tumor-bearing mice, the animals were briefly placed in a restraining device and injected via the tail vein with a 0.1 mmol Fe/kg (naïve mice) or a 0.3 mmol Fe/kg (tumor-bearing mice) dose of the iron chelate agent. Gadopentetate dimeglumine (**Gd-1**; Magnevist™, Bayer) was used in both naïve and tumor imaging studies as the GBCA control agent, given at the same dosage as the iron chelates. During imaging, animals were anesthetized with 2-3% isoflurane carried in medical grade oxygen. All animals were imaged on a GE Healthcare Sigma 1.5 T scanner (Milwaukee, WI). A precontrast T_1_ scan (fSPGR, TR: 150 ms, TE: 3.9 s, FA: 90, NEX: 5) and T_2_ scan (FSE XL, TR: 4000 ms, TE: 90.1 ms, FA: 90, NEX: 1) were obtained for each animal before injection. Multiple transaxial slices were acquired through the heart, liver, kidney, tumor (for tumor-bearing mice), and bladder. A custom-made transmit/receive mouse body solenoid coil was used; animals were positioned prone, and tumors (for tumor-bearing mice) were consistently positioned at the centerline of the coil. Animals were then imaged again at 5, 10, 15, and 30 minutes after injection using the same imaging parameters. An external phantom consisting of a glass tube filled with corn oil was also placed in the coil with the animal, such that a circular cross section of the phantom was visible in all acquired slices.

#### 2.3.2. Image Analysis

Image data were analyzed using CineTool (Version 8.5.0; GE Healthcare), which is a custom, research-use-only image analysis software toolkit running on IDL Virtual Machine (Version 6.3) [32]. Regions-of-interest (ROIs) were manually annotated using CineTool to encompass the kidney cortex, kidney medulla, liver, and external phantom for all animals studied. For tumor-bearing mice, ROIs of non-necrotic tumor, necrotic tumor (identified using T_2_-weighted images), and muscle were additionally drawn. ROIs for a given tissue were typically drawn on 3 sequential slices in Z. Average signal values within all drawn ROIs at all time points were exported from CineTool as a text file and manipulated in Microsoft Excel as follows. First, all ROI data points were normalized to the corresponding external phantom ROI from the same image. Normalization of signal to the external phantom corrects for possible drift in the image intensity scaling across exams due to gain settings, shimming, and positioning within the solenoid coil. Second, the normalized ROI values for each tissue were averaged across multiple slices at each time point, weighted according to the area of the ROI on each image, to provide an average volumetric intensity value for each tissue at each time point. Third, these time-series data were used to calculate signal enhancement (SE) = (signal postinjection)/(signal preinjection) at each time point. Finally, the SE was then averaged across mice for each contrast agent group. Tests for statistical significance were run with Minitab v18.1, using ANOVA with a significance level taken at *p* < 0.05. Interval charts of imaging data are shown with 95% confidence intervals calculated using the pooled standard deviation.

## 3. Results and Discussion

### 3.1. Rationale for Analog Selection

Fe(III) chelates investigated in this report are shown in Figure 1. As described in the Introduction, Fe-HBED (**Fe-1**) is an attractive parent chelate for an IBCA; the goal of this work was to modify HBED to improve MRI signal and reduce serum protein binding as well as liver signal relative to Fe-HBED.

#### 3.1.1. Improving MRI Signal

T_1_-weighted image contrast from MRI CA depends on the ability of the CA to shorten the longitudinal relaxation time of protons on nearby water molecules; this capability is typically reported as *r*_1_ relaxivity and given in units of mM^−1^·s^−1^. The measured relaxivity represents contributions from 3 different types of water protons, as shown in the following equation [20, 33, 34]:

(1) $$\begin{array}{c} r_{1}=r_{1}^{\text{IS}}+r_{1}^{\text{SS}}+r_{1}^{\text{OS}}, \end{array}$$

where *r*_1_^IS^ inner sphere relaxivity arising from water molecules coordinated to the metal ion, *r*_1_^SS^ is the second sphere relaxivity arising from water molecules interacting with the ligand, and *r*_1_^OS^ is the outer sphere relaxivity arising from bulk water molecules.

The challenge with Fe chelates is that the Fe must be coordinatively saturated to minimize redox chemistry. Consequently, there is no available site to which water may coordinate directly to iron, and therefore, inner sphere relaxivity is precluded. Improving second sphere ligand-water interactions represents the most significant mechanism to leverage in order to improve IBCA relaxivity.

Increasing second sphere hydration by incorporating polar groups is effective in increasing relaxivity of Gd chelates with an open coordination site for water; it is not widely exploited because of the predominant contribution of inner sphere relaxivity available for coordinatively unsaturated Gd complexes [33, 35–37]. It seemed reasonable to postulate that adding hydrophilic sites to the ligand with which water can interact may improve second sphere relaxivity of coordinatively saturated IBCA; this was done in **Fe-2–Fe-5** in Figure 1.

#### 3.1.2. Reducing Serum Protein Binding

Fe-HBED (**Fe-1**) is taken up and cleared primarily via the liver which makes it of interest as a liver MRI CA but not as a more widely applicable ECF MRI CA [28–30]. In order to reduce nonspecific hydrophobic interactions with serum proteins and reduce liver uptake, the hydrophilicity of the parent HBED chelate was increased. The efficacy of this approach has already been demonstrated for HBED (**1**) and the more polar analog SHBED (**2**) as their complexes with ^111^In; ^**111**^**In-2** showed lower liver uptake and higher kidney/bladder uptake relative to ^**111**^**In-1** [38]. The bisphosphonate analog, HBEDP (**3**), has been reported to form a stable complex with Fe[III] [39]. Phosphonic acids are more polar isosteres of carboxylic acids [40, 41], and the log *P* of a series of aromatic phosphonic acids was one log *P* unit lower than the corresponding carboxylic acids [42]. It was hypothesized that the phosphonate groups would increase the chelate hydrophilicity and reduce binding to serum proteins, leading to improved renal clearance. HBEDP analogs **4** and **5** were functionalized with hydroxyl groups, to further increase hydrophilicity in anticipation that the corresponding Fe[III] chelates, **Fe-4** and **Fe-5**, would show even less liver uptake.

Unfortunately, it is expected that reducing serum protein binding will also reduce *r*_1_ because of increased molecular tumbling in solution. When a CA is bound to a protein, tumbling or rotational correlation time of the CA is reduced; this will contribute to an increase in *r*_1_ [34]. Indeed, this approach is often used to increase *r*_1_, at the expense of ECF behavior [20]. Because this work focuses on developing a Fe chelate that behaves as an ECF MRI CA, the analogs in Figure 1 were selected based on the rationale that incorporating hydrophilic groups would reduce serum protein binding while also increasing *r*_1_ due to increased second sphere hydration. In addition, synthetic accessibility was a guiding consideration in the selection of target analogs.

### 3.2. Synthesis

**Figure 2:**
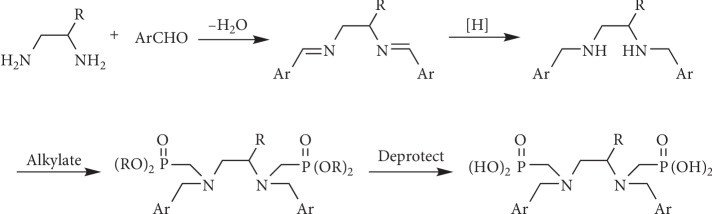
General synthetic scheme for HBEDP analogs **3**, **4**, and **5**.

SHBED was prepared as previously described [38]. The bisphosphonate ligands (3–5) were prepared according to the general scheme in Figure 2. Synthetic details are provided in the supplemental material.

### 3.3. Relaxivity

The modified analogs of Fe-HBED (**Fe-1**) showed improved *r*_1_ relaxivity ranging from 2-3*x* greater than the HBED parent, with the phosphonic acid analogs Fe-HBED (**Fe-3**) and Fe-HBED-(CH_2_OH)_3_ (**Fe-5**) showing the largest increase (Table 1). Analog **Fe-4** is a good demonstration of the complicated structure-activity relationship for *r*_1_ relaxivity; despite having 2 additional hydroxyl groups, the relaxivity of **Fe-4** is inferior to the closely related HBEDP (**Fe-3**) that lacks hydroxyl groups. It is also noteworthy that *r*_2_/*r*_1_ values for the Fe chelates are comparable to clinically approved GBCA at 1.5 T in water (1.07–1.19) [43]. This similarity allows the IBCA to match T_1_ and T_2_ relaxation rates of GBCA simply by increasing the concentration of the IBCA, as appropriate.

### 3.4. Redox Stability

**Figure 3:**
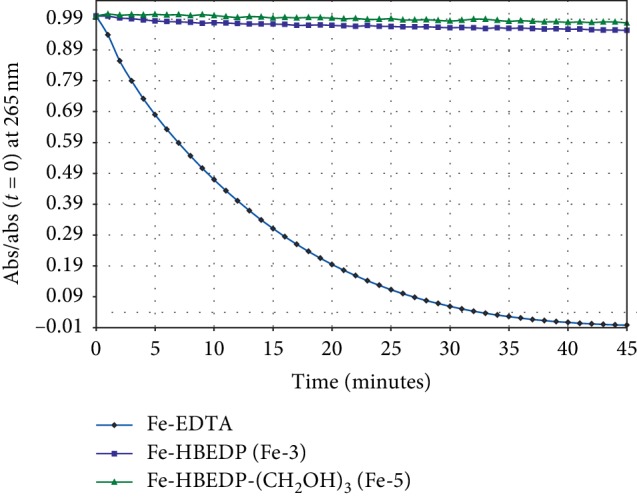
Air oxidation of ascorbic acid catalyzed by **Fe-3** and **Fe-5** and positive control Fe-EDTA.

The potential for iron-catalyzed Fenton chemistry is a risk that must be considered for IBCA. Based on the previously reported redox inertness of Fe-HBED, **Fe-1** [22–24], it was expected that analogs **Fe-2** through **Fe-5** would likewise be redox inert. This was confirmed by measuring the Fe chelates' ability to catalyze the oxidation of ascorbic acid.  Figure 3 shows the consumption of ascorbic acid by iron catalyzed air oxidation for **Fe-3**, **Fe-4,** and positive control Fe-EDTA. The pseudo-first-rate time constants for ascorbic acid consumption for **Fe-2** (data not shown), **Fe-3,** and **Fe-5** are <0.5% that of Fe-EDTA. Oxidative catalytic inertness is a promising preliminary sign for these Fe-HBED analogs, but further development as MRI CA would require toxicity and safety studies, including long-term chelate stability and histologic studies.

### 3.5. Serum Protein Binding

Binding to serum proteins slows the extravasation of chelates and slows renal filtering, thereby extending plasma half-life and increasing hepatic clearance. Fe-HBED shows significant association with serum proteins and has, thus, been investigated as a liver-specific MRI CA because of significant liver uptake [28–30]; this makes it unsuitable as an ECF agent. To reduce protein association of Fe-HBED, hydrophilic substituents were incorporated in the HBED ligand. This strategy is illustrated by sulfonate derivatives, Fe-SHBED (**Fe-2**), which showed no appreciable serum protein binding. Interestingly, as shown in Table 1, exchanging the carboxylates in Fe-HBED (**Fe-1**) with the phosphonates in Fe-HBEDP (**Fe-3**) did not reduce protein binding. This was unexpected as phosphonates are considered to be more polar isosteres of carboxylic acids [40–42]; however, the greater negative charge of Fe-HBEDP (**Fe-3**) relative to Fe-HBED (**Fe-1**) may increase interaction with the positively charged binding pocket of albumin [44]. The Fe chelates of HBEDP analogs **4** and **5,** which were further functionalized with hydroxyl groups, showed reduced protein binding relative to both **Fe-1** and **Fe-3**.

### 3.6. Imaging in Mice

The performance of all five iron chelate agents was evaluated in mouse imaging studies of the kidney and liver, with both organs imaged simultaneously in a multislice acquisition. The kidney was selected as an imaging target largely as a surrogate for blood volume enhancement acquired with minimal flow artifact present in larger vessels, owing to the dense, nonisotropic capillary beds in the renal cortex. Significant liver enhancement, while also due in part to the circulating agent within a high fractional blood volume of the organ, is generally attributable to uptake of contrast agent by hepatocytes and/or Kupffer cells, the latter being generally more relevant in the case of nanoparticulate or opsonized contrast agents. As discussed above, binding of a contrast agent to serum proteins extends circulation time and increases the proportion of uptake of the contrast agent in the liver.

The reported studies occurred over a project period of approximately two years, and in that time, some changes to the study protocol were implemented. In particular, **Fe-1** and **Fe-2** were initially evaluated in naïve mice, whereas later in the project time course, liver and kidney imaging data for **Fe-2**, **Fe-3**, **Fe-4**, and **Fe-5** were acquired from tumor-bearing mice. This change was implemented as a refinement to reduce the number of animals necessary to complete the project aims of both organ-specific and tumor imaging. In both study types, gadopentetate dimeglumine (**Gd-1**) served as the GBCA comparator. Images were acquired at 5, 10, 15, and 30 minutes; the results are shown at 5 minutes when differences between agents were greatest, though similar trends were observed at later times.

### 3.7. Evaluation of **Fe-1** and **Fe-2** in Naïve Mice

**Figure 4:**
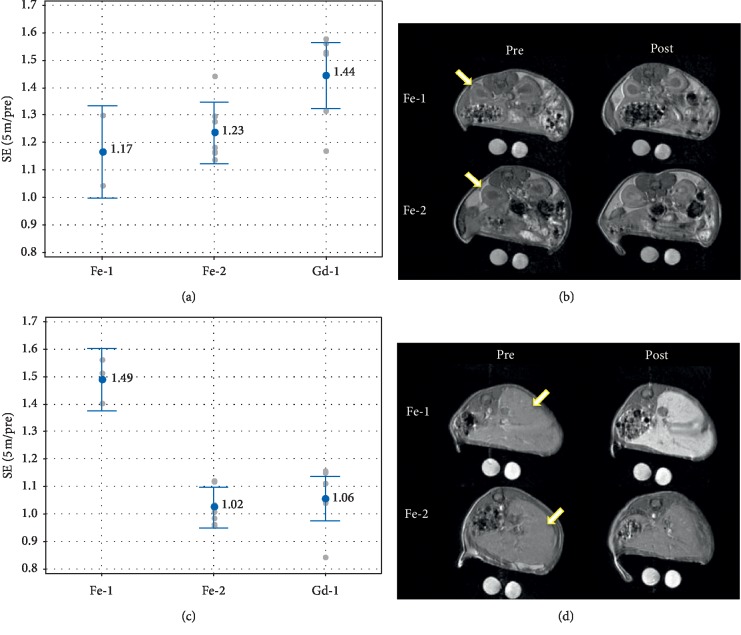
Tissue signal enhancement of contrast agents in the kidney cortex and liver in naïve mice. Quantified SE is given for **Fe-1**, **Fe-2**, and **Gd-1** in the kidney cortex (a) and in the liver (c). 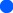 Mean with value; 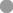 individual data points. Data labels are the mean. Corresponding images of preinjection (left) and 5 minutes postinjection (right) for the mouse representing median enhancement for each agent group for the kidney (b) and liver (d). The window and level of the images are scaled for uniformity. Arrows denote the left kidney cortex (b) and liver (d).

In the left kidney cortex (Figure 4(a)), signal enhancement (SE, see experimental section for derivation) afforded by **Gd-1** (*r*_1_ = 3.3 mM^−1^·s^−1^ in water at 1.5 T [43]) is greater (*p* > 0.05) than either iron chelate, consistent with the relaxivity of the contrast agents. The SE afforded by **Fe-2** (*r*_1_ = 0.9 mM^−1^·s^−1^) and **Fe-1** (*r*_1_ = 0.49 mM^−1^·s^−1^) are similar (*p*=0.74) despite the greater *r*_1_ of **Fe-2**, presumably because of the higher protein binding of **Fe-1** which serves to increase relaxivity in vivo.

In the liver, however, the SE afforded by **Fe-1** is much greater than **Fe-2** and **Gd-1** (*p* < 0.0001) even though **Fe-1** has the lowest *r*_1_; this is presumably due to increased uptake in the liver, consistent with literature evaluation of **Fe-1** as a liver-specific agent [28–30]. The SE of **Gd-1** and **Fe-2** are consistent with their relaxivities but the difference is not significant (*p*=0.81). This shows that adding polar groups to liver-specific CA **Fe-1** yields a contrast agent, **Fe-2,** with liver uptake comparable to ECF GBCA **Gd-1**.

### 3.8. Evaluation of **Fe-2**, **Fe-3**, **Fe-4**, and **Fe-5** in a Mouse Tumor Model

**Figure 5:**
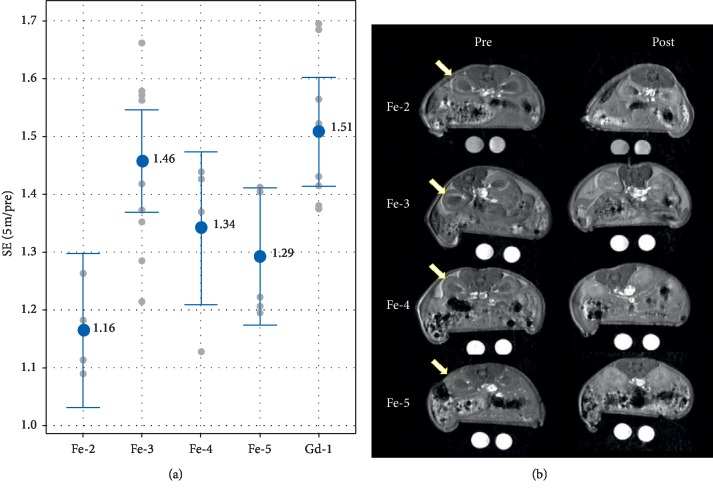
Signal enhancement in the kidney cortex evaluated in the tumor mouse model. (a) SE (5 min/pre) of **Fe-2** to **Fe-5** and **Gd-1** in the left kidney cortex. 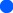 Mean; 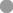 individual data points. Data labels are the mean. The SE afforded by **Gd-1** is greater than **Fe-2** and **Fe-5** (*p* ≤ 0.05). **Fe-3** SE is greater than **Fe-2** SE (*p*=0.007). (b) Preinjection (left) and 5 minutes postinjection (right) images for the mouse representing median enhancement for each agent group. The window and level of the images are scaled for uniformity. Arrows denote the left kidney cortex.

Figure 5 shows the kidney signal enhancement and corresponding images obtained in the tumor model. Results are consistent with the data in Table 1 in that **Fe-3** has both a relatively high *r*_1_ relaxivity and the greatest degree of protein binding; both of these factors should lead to the greatest relaxivity in vivo. **Fe-4** and **Fe-5** have an intermediate level of protein binding and relatively high *r*_1_ relaxivities in PBS, and they appear to show an intermediate signal level in the kidney cortex, whereas **Fe-2** has a relatively lower signal in the kidney consistent with its moderate relaxivity and low protein binding. Based on the observations in Figures 4 and 5, it is reasonable to conclude that the net signal in the renal cortex, which is taken as a reflection of signal in circulating blood, is attributed to the combination of both the inherent chelate relaxivity and its degree of protein binding.

**Figure 6:**
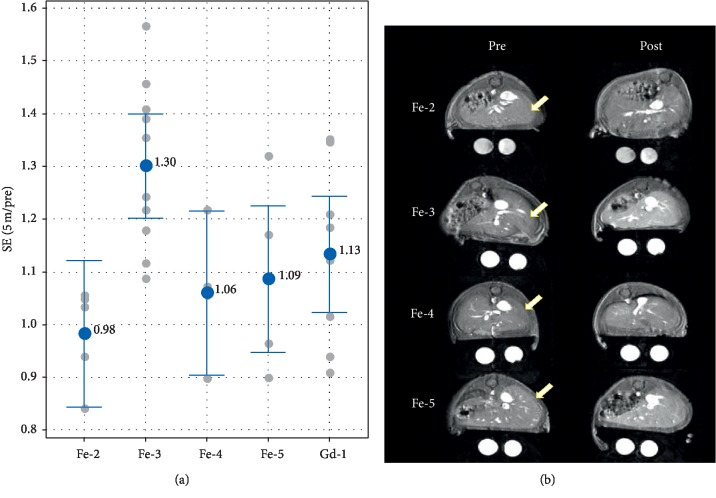
Performance of the iron agents in imaging of the mouse liver. (a) Enhancement ratios (post/pre) of the 4 agents in the liver. **Fe-3** is significantly different than **Fe-2** (*p*=0.006), and other differences are not significant (*p* > 0.1). 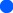 Mean ± 95% confidence interval; 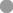 individual data points. (b) Preinjection (left) and 5 minutes postinjection (right) images for the mouse representing median enhancement for each agent group. The window and level of the images are scaled for uniformity. Arrows denote the liver regions used for analysis.

While these trends defined in the kidney cortex are expected to also remain true while agents reside in the general circulation within the liver organ, the liver may additionally develop signal from the accumulation of agent via cellular uptake. Furthermore, the degree of liver uptake is in part dependent on the degree of plasma protein binding during circulation in the blood. Therefore, as opposed to enhancement in the kidney cortex above, these combined signal effects of protein binding are expected to be more dominant in the liver. Consistent with this, Figure 6 shows that **Fe-3** with 49% assay protein binding exhibited the greatest SE in liver tissue, whereas **Fe-2** (0% protein binding) had essentially no SE in the liver. **Fe-4** and **Fe-5**, each with 20% assay protein binding, were also relatively low in liver signal. Together with Figure 4(c), this shows that adding polar groups to liver-specific CA **Fe-1** yields contrast agents, **Fe-2**, **Fe-4**, and **Fe-5**, with liver SE levels that are comparable to ECF GBCA, **Gd-1**.

**Figure 7:**
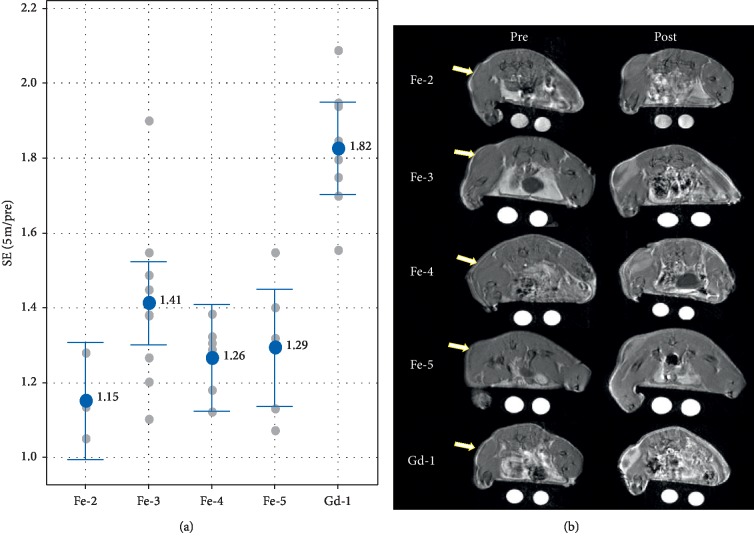
Performance of the iron agents in imaging of the C6 glioma tumor model. (a) Enhancement ratios (post/pre) of 4 iron chelate agents. There is no significant difference in the means of tumor enhancement for the iron chelates (*p* > 0.06), though **Fe-3** trended higher, as was also the case for the kidney cortex. The differences between **Gd-1** and all the iron chelates were significant (*p* < 0.001). 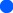 mean ± 95% confidence interval; 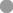 individual data points. (b) Preinjection (left) and 5 minutes postinjection (right) images for the tumor-bearing mouse representing median enhancement for each agent group. The window and level of the images are scaled for uniformity. Arrows indicate the position of the subcutaneous tumor.

As an initial exploration of the potential utility of iron chelates for detection and characterization of solid tumors, agents **Fe-2**, **Fe-3**, **Fe-4**, **Fe-5**, and **Gd-1** were evaluated in a glioma tumor model grown subcutaneously in nude mice.  Figure 7 shows results of the 5-minute post-injection time point. Although there was no statistical difference in the mean enhancement values for the four iron agents, the groups appeared to trend similarly to the results shown above for the kidney cortex. Since the tumor signal is driven by the presence of agent both in the blood volume and extravasated into the extracellular space of the tumor, it is not surprising that the agents should perform similarly to their relative performance in the kidney cortex. The mean enhancement values for all of the iron agents were significantly lower than that for the **Gd-1** GBCA comparator. Although the magnitude of the observed tumor enhancement of roughly 20% to 40% across the iron agents is moderate and roughly half that of GBCA at the same dose (0.3 mmol metal/kg), it is worth noting that the overall signal of the iron chelates could be increased in future studies by increasing the dose, which is reasonable to contemplate given the favorable toxicity profile of iron in the context of these Fe chelates that do not catalyze Fenton chemistry.

## 4. Conclusions

Analogs of iron chelate Fe-HBED were prepared and evaluated as ECF MRI-CA. Modifications in the ligand structure improved Fe chelate relaxivity and reduced serum protein binding while maintaining the redox inertness of Fe chelated to HBED. MRI in mice showed useful contrast of vasculature and tumor tissue. Liver signal enhancement afforded by the Fe-HBED analogs was reduced relative to the parent Fe-HBED to a level comparable to that of an ECF GBCA; this is a key feature of ECF agents. This work establishes that the HBED ligand framework is a viable platform for the development of next-generation ECF CA for MRI that does not use Gd metal.

## Figures and Tables

**Table 1:** Relaxivity and protein binding of Fe chelates.

ID	Abbreviation	*r*_1_ (mM^−1^·s^−1^)	*r*_2_ (mM^−1^·s^−1^)	*r*_2_/*r*_1_	Protein binding (%)
**Fe-1**	Fe-HBED	0.49	0.52	1.06	33
**Fe-2**	Fe-SHBED	0.9	1.0	1.15	0
**Fe-3**	Fe-HBEDP	1.3	1.6	1.20	49
**Fe-4**	Fe-HBEDP-(CH_2_OH)_2_	0.9	1.2	1.33	20
**Fe-5**	Fe-HBEDP-(CH_2_OH)_3_	1.5	1.7	1.13	20

## Data Availability

The data used to support the findings of this study are included within the article and within the supplementary information files.

## Abbreviations

CA: Contrast agent
ECF: Extracellular fluid
Fe: Iron
GBCA: Gadolinium-based contrast agent
Gd: Gadolinium
HBED: *N,N*′-bis(2-hydroxyphenyl)ethylenediamine-*N,N*′-diacetic acid
IBCA: Iron-based contrast agent
MRI: Magnetic resonance imaging
PBS: Phosphate-buffered saline
ROI: Region of interest
SE: Signal enhancement.

## Supplementary Materials

Supplementary Materials Supplementary Materials contain details on the synthesis of analogs **Fe-3**, **Fe-4**, and **Fe-5**, imaging data in a tabular form and results from statistical tests of imaging data.
