# Supplementary material for: Fe-HBED Analogs: A Promising Class of Iron-Chelate Contrast Agents for Magnetic Resonance Imaging
Source: Contrast Media Mol Imaging. 2019 Dec 20;2019:8356931. doi: 10.1155/2019/8356931 (PMC6961518; doi:10.1155/2019/8356931)
Supplement: Supplementary Materials — contain details on the synthesis of analogs Fe-3, Fe-4, and Fe-5, imaging data in a tabular form and results from statistical tests of imaging data. [file 8356931.f1.pdf]

Fe-HBED Analogs – A promising class of iron-chelate contrast agents for magnetic resonance imaging

Contents

Chemical Synthesis ... 1
General Procedures. ... 1
Compounds Fe-1 and Fe-2 ... 1
Preparation of ligand HBEDP (3) and iron complex Fe-HBEDP (Fe-3) ... 2
Preparation of ligand HBEDP-$(CH_2OH)_2$ (4) and iron complex Fe-HBEDP-$(CH_2OH)_2$ (Fe-4) ... 3
Preparation of iron complex Fe-HBEDP-$(CH_2OH)_3$ (Fe-5) ... 4
Imaging Data ... 6
Naïve model ... 6
Kidney Cortex ... 6
Liver ... 7
Tumor model ... 7
Kidney cortex ... 7
Liver ... 8
Tumor ... 8
References ... 9

Chemical Synthesis

General Procedures.

Reactions requiring a dry environment were performed under a nitrogen atmosphere in glassware dried at 150 °C prior to use; anhydrous solvents were obtained through standard laboratory protocols. Melting points were obtained on an Unimelt™ Thomas Hoover capillary melting point apparatus and are not corrected. Analytical thin-layer chromatography (TLC) was performed on SiO_2 60 F-254 plates available from Merck using the mobile phase indicated. Visualization was accomplished by UV irradiation at 254 of 304 nm, or by staining with one of the following reagents: 5% phosphomolybdic acid hydrate in ethanol (PMA), ninhydrin (0.3% w/v in glacial acetic acid/*n*-butanol 3:97), or vanillin (5% w/v in concentrated H_2SO_4/ethanol 1:99) stain. Flash chromatography was performed on a CombiFLASH Companion™ using 4, 12, 40, or 120 g SiO_2 columns or reversed phase C_{18} columns and eluants were monitored at the wavelengths indicated. Proton and carbon NMR spectra were obtained on Brucker Avance 400 and 500 MHz NMR spectrometers. Chemical shifts are reported in parts per million (ppm) against the solvent residual of the NMR solvent employed (1). NMR peak multiplicities are denoted as follows: s (singlet), d (doublet), t (triplet), q (quartet), p (pentet), bs (broad singlet), dd (doublet of doublet), tt (triplet of triplet), ddd (doublet of doublet of doublet), and m (multiplet). Coupling constants (*J*) are given in hertz (Hz). High resolution mass spectra (HRMS) were obtained using the indicated ionization mode by the GE Global Research Analytical Sciences Laboratory. *N*,*N*'-Di(2-hydroxybenzyl)ethylenediamine-*N*,*N*'-diacetic acid (HBED) • HCl, and Fe-HBED (CAS#16455-61-1) were purchased from commercial vendors. *N*,*N*'-Di(2-hydroxy-5-sulfobenzyl)ethylenediamine-*N*,*N*'-diacetic acid (SHBED) was synthesized in accordance with previously published procedures.(2)

Compounds Fe-1 and Fe-2

A solution of $FeCl_3{\cdot}6\ H_2O$ (1.1 eq.) was added to a mixture of the metal free ligand (HBED (**1**) or SHBED **2**) in water (~0.1 M) The reaction mixture was stirred until a homogeneous or nearly homogeneous solution was formed. The pH of the solution was adjusted to ~5 by adding *N*-methyl-D-glucamine. The resulting precipitate was collected by centrifugation, washed with water, and subsequently resuspended in water. Following this, *N*-methyl-D-glucamine was added to adjust the pH of the solution to 9 yielding the

compounds **Fe-1** or **Fe-2** as homogeneous red solutions. The solutions were assayed for total Fe concentration, and measurements of r_1 and r_2 values were obtained. The purity and identity of the product was further confirmed by HPLC-MS analysis.

Preparation of ligand HBEDP (3) and iron complex Fe-HBEDP (Fe-3)

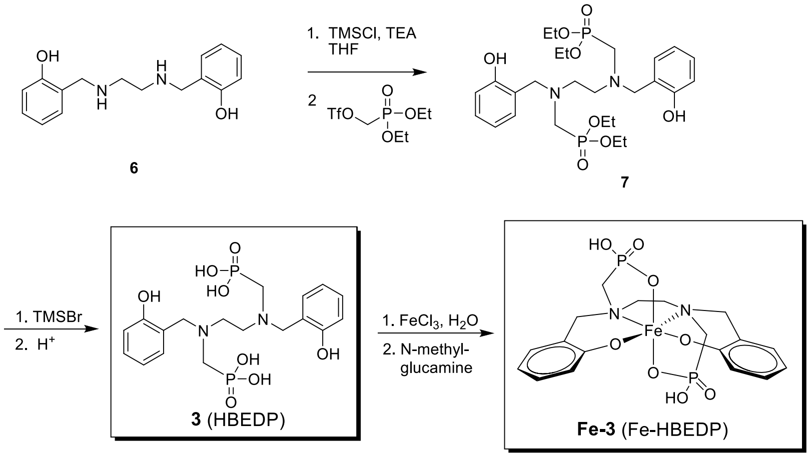

Compound 7. To a solution of 2,2'-(bis (2-hydroxybenzyl) ethylene diamine **6** (0.25 g, 0.92 mmol) in 5 milliliters (mL) of anhydrous tetrahydrofuran (THF) at 0 °C was added triethylamine (TEA) (0.63 mL, 4.6 mmol) followed by the addition of 0.23 mL (2.0 mmol) of trimethylsilyl chloride (TMSCl). The reaction mixture was stirred for 30 minutes. A solution of phosphonomethyltriflate (0.67 gram, 2.0 mmol) in 1 mL of THF was added to the reaction mixture. The reaction mixture was stirred overnight, slowly warming to room temperature over this time. The mixture was poured into saturated aqueous $NaHCO_3$ and diluted with 20 mL of diethylether (Et_2O). The aqueous and organic layers were separated and the aqueous layer was extracted with Et_2O (3 x 25 mL). The combined organic layers were washed with saturated aqueous $NaHCO_3$, (2 x 25 mL), and brine (2 x 25 mL), dried over $MgSO_4$ and filtered. The filtrate was concentrated under reduced pressure to provide the crude product as a pale yellow oil. The crude product was purified by flash chromatography on normal phase silica (SiO_2, 12g) using the following gradient program at 30 mL/min: 2% MeOH-CH_2Cl_2 for 5 column volumes, then ramp to 10% MeOH-CH_2Cl_2 over 30 column volumes, finally holding at 10% MeOH-CH_2Cl_2 for 5 column volumes. The column eluant was monitored at 277 nm and the purified material was pooled and concentrated under reduced pressure to provide compound **7** as a colorless oil that was further dried under high vacuum (80 % yield) and analyzed using liquid chromatography–mass spectrometry–electrospray ionization (LCMS (ESI)) 595 $(M+Na)^+$.

Compounds 3 and Fe-3. To a solution of **7** (0.42 g, 0.74 mmol) in 7.4 mL of dichloromethane was added 0.78 mL, (5.9 mmol) of TMSBr at room temperature. The reaction mixture was heated to 75 °C for 120 min to allow for clean conversion to the product **3** (HBEDP) as evidenced by LCMS ESI 461 $(M+H)^+$. The solvent was removed under reduced pressure, and the residue was diluted with acetone-H_2O (4:1) and stirred overnight. The remaining solvent was removed under reduced pressure and the residue was dissolved in water. Ferric chloride ($FeCl_3$ $6H_2O$, 0.93 equivalents) solution was added to the residue followed by addition of 1 molar (M) NaOH to adjust the pH of the solution to 7.4. The solution was filtered through a Sephadex G-10 column to yield a filtrate containing the complex **Fe-3** (FE-HBEDP) in which the charge balancing counterion Q is believed to be primarily sodium cation. The filtrate was subsequently assessed for total Fe concentration and relaxivity, LCMS (ESI) 513 $(M+H)^+$ λ_{max} (DI) = 455nm.

Preparation of ligand HBEDP-$(CH_2OH)_2$ (4) and iron complex Fe-HBEDP-$(CH_2OH)_2$ (Fe-4)

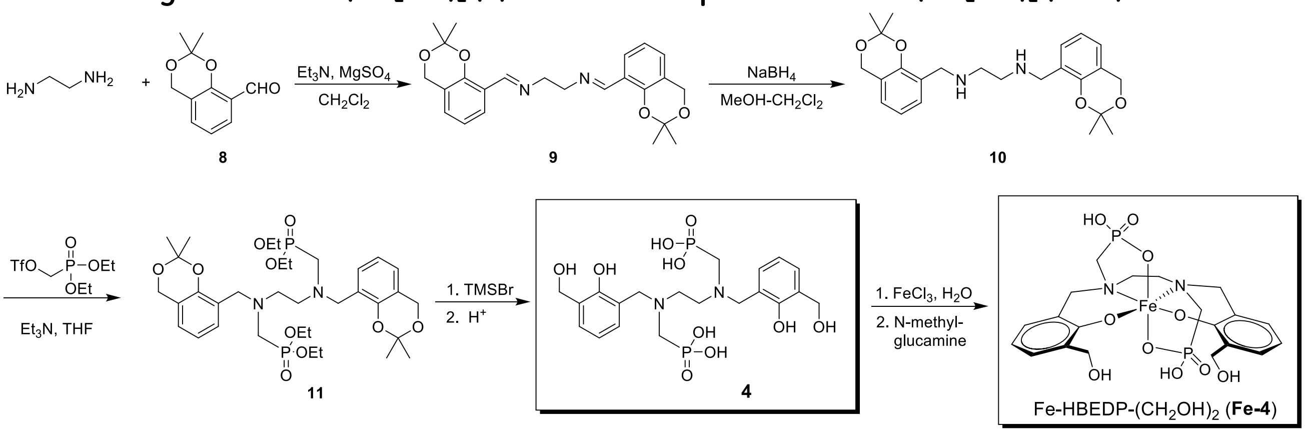

Compound 9. To ethylenediamine (126 µL, 1.88 mmol) in (5 mL) was added triethylamine (TEA) (654 µL, 4.69 mmol) followed by $MgSO_4$ (903 mg, 7.5 mmol) and the resultant mixture was stirred for 1.5 h at room temperature. The aldehyde **8** (721 mg, 3.75 mmol) in CH_2Cl_2 (3 mL) was then added and the reaction mixture was stirred overnight. The reaction mixture was filtered and then concentrated under reduced pressure to provide the crude bisimine **9** containing a small quantity of unreacted aldehyde. The conversion of aldehyde to imine was confirmed by NMR spectroscopy: 1H NMR (CD2Cl2, 400 MHz) δ 1.54 (s, 12H), 3.94 (s, 4H), 4.87 (s, 4H), 6.92 (t, J = 8 Hz, 2H), 7.05 (d, J = 8 Hz, 2H), 7.82 (d, J = 8 Hz, 2H), and 8.64 (s, 2H).

Compound 10. To a solution of bisimine **9** (700 mg, 1.71 mmol) in dichloromethane (6.8 mL) and methanol (1.7 mL) at 0 °C was added $NaBH_4$ (259 mg, 6.85 mmol). The reaction mixture was allowed to stir overnight while slowly warming to room temperature and was then diluted with saturated aqueous Na_2CO_3. The aqueous and organic layers were separated. The aqueous layer was extracted with CH_2Cl_2 (3 x 25 mL). The combined organic layers were washed with saturated aqueous $NaHCO_3$ (2 x 25 mL) and brine (2 x 25 mL), dried over Na_2SO_4 and filtered. The filtrate was concentrated under reduced pressure to provide the crude product **10** as a pale yellow oil. The crude product **10** was purified by flash chromatography on normal phase silica gel (40 gram column) using the following gradient program at 40 mL/min: 100% CH_2Cl_2 containing 0.5% triethylamine (TEA) for 3 column volumes, then ramp to 5% MeOH-CH_2Cl_2 each containing 0.5% TEA over 20 column volumes, finally holding at 5% MeOH-CH_2Cl_2 each containing 0.5% TEA for 5 column volumes. The column eluant was monitored at 285 nm and fractions containing the purified material were combined, concentrated under reduced pressure and dried under high vacuum to yield the purified compound **10** as a colorless oil. The purified compound **10** was analyzed by NMR spectroscopy and mass spectrometry. ^1H NMR (CD_2Cl_2, 400 MHz) δ 1.57 (s, 12H), 1.86 (br s, 2H), 2.73 (s, 4H), 3.78 (s, 4H), 4.88 (s, 4H), 6.88-6.94 (m, 4H), and 7.19 (m, 2H); *m/z* = 414 $[M+H]^+$.

Compound 11. To a solution of diamine compound **10** (486 mg, 1.18 mmol) in anhydrous THF (12 mL) at 0 °C was added TEA (658 µL, 4.72 mmol) followed by dropwise addition of (diethoxyphosphoryl)methyl trifluoromethanesulfonate (1.08 g, 3.60 mmol). The reaction mixture was allowed to warm to room temperature and stirred overnight. The reaction mixture was then quenched with saturated aqueous $NaHCO_3$. The aqueous and organic layers were separated and the aqueous layer was extracted with CH_2Cl_2 (3 x 25 mL). The combined organic layers were washed with saturated aqueous $NaHCO_3$ (2 x 25 mL) and brine (2 x 25 mL), dried over Na_2SO_4 and filtered. The filtrate was concentrated under reduced pressure to provide the crude product as a pale yellow oil which was purified by flash chromatography on normal phase silica gel (40 gram column) using the following gradient program at 40 mL/min: ramp from hexanes containing 0.5% TEA to 75% EtOAc-hexanes each containing 0.5% TEA over 2 column volumes, then ramp to 95% EtOAc-hexanes each containing 0.5% TEA over 13 column volumes, finally holding at

95% EtOAc-hexanes each containing 0.5% TEA for 10 column volumes. The column eluant was monitored at 285 nm and fractions containing the purified material were combined and concentrated under reduced pressure to yield purified compound **11** as a colorless oil after drying under high vacuum. The structure of compound **11** was confirmed by NMR spectroscopy and LCMS. ^{1}H NMR (CD_2Cl_2, 400 MHz) δ 1.29 (t, J = 8 Hz, 12H), 1.53 (s, 12H), 2.87 (s, 4H), 2.94 (d, J = 8 Hz, 4H), 3.75 (s, 4H), 4.03-4.12 (m, 8H), 4.85 (s, 4H), 6.86-6.91 (m, 4H), and 7.30 (d, J = 8 Hz, 2H); LCMS *m/z* = 714 $[M+H]^+$, 736 $[M+Na]^+$.

Compound 4 (HBED-$(CH_2OH)_2$). To a stirred solution of compound **11** (157 mg, 0.22 mmol) in of anhydrous CH_2Cl_2 (7.0 mL) and anhydrous CH_3CN (7.0 mL) was added bromotrimethylsilane (0.40 mL, 3.09 mmol) at room temperature. The reaction mixture was then heated at 50 °C for 30 hours. The solvent was removed under reduced pressure and the residue was stirred overnight in an acetone:water mixture (4:1 v/v) at room temperature. The resulting suspension was subjected to centrifugation and the precipitate was washed with water and acetone to afford ligand **4** as a colorless solid which was used immediately to prepare **Fe-4** as described below.

Compound Fe-4 (Fe-HBED-$(CH_2OH)_2$). Ligand **4** prepared as described above, was suspended in 3 mL of water H_2O was stirred at 50 °C and progress of the reaction was monitored by LCMS. Upon completion of the reaction, the pH of the reaction mixture was adjusted to 5 by the addition of *N*- methyl-D-glucamine. Iron complex **Fe-4** was obtained as a precipitate which was collected by centrifugation, washed twice with water, and then resuspended in water. Additional *N*-methyl-D-glucamine was then added to adjust the pH to 9. The resulting red solution was filtered through a 0.1 μm syringe filter and analyzed by LCMS to confirm the presence of **Fe-4**, m/z = 572 $[M]^+$, λ_{max} (DI) = 465nm.

Preparation of iron complex Fe-HBEDP-$(CH_2OH)_3$ (Fe-5)

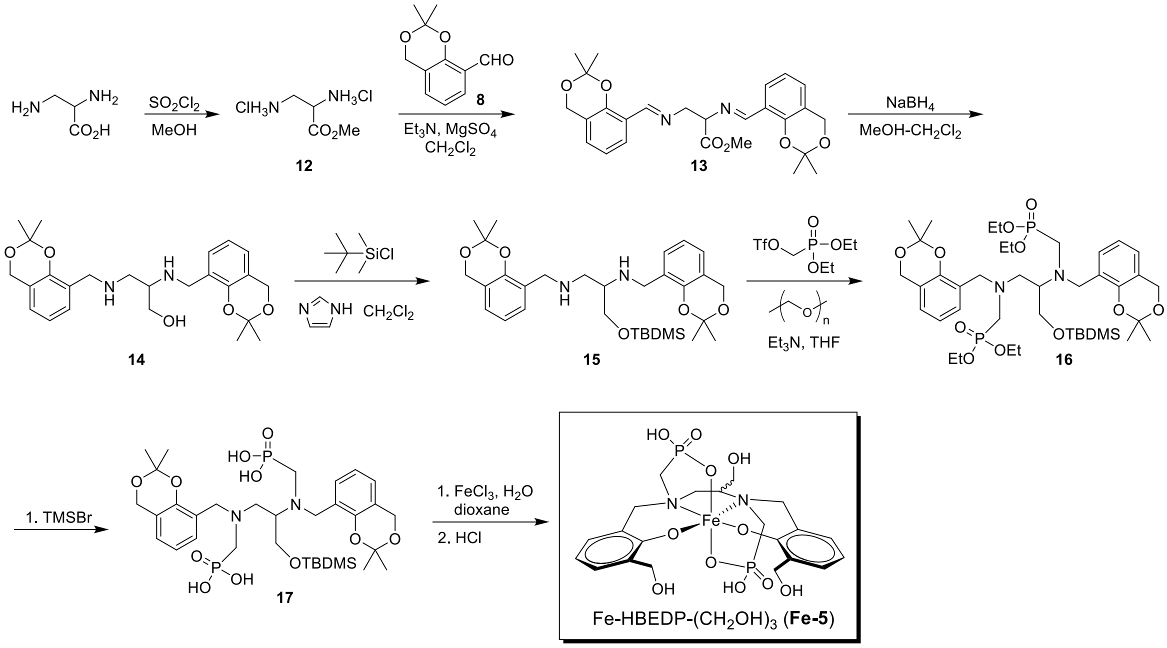

Compound 12. Thionyl chloride (31.7 g, 266.8 mmol) was added drop wise to a stirred suspension of 2,3-diaminopropionic acid monohydrochloride (5.0 g, 35.6 mmol) in MeOH (75 mL) over a period of 5 min. The reaction mixture was heated to 80 °C for 6 h. The reaction mixture was then cooled and the volatiles were removed under reduced pressure to obtain compound **7** (6.8g, 100%) as an off-white solid. ^{1}H NMR (MeOD): δ 4.51(m, 1H), δ 3.96 (s, 3H), δ 3.53 (m, 2H).

Compoune 13. To a suspension of the diamine compound 12 (1.00 g, 5.2 mmol) in CH_2Cl_2 (15 mL) at room temperature was added TEA (3.3mL, 23.6 mmol) and $MgSO_4$ (2.5 g, 20.9 mmol). The reaction mixture was

stirred for 1.5 h at room temperature and then a solution of the aldehyde **8** (2.0 g, 10.6 mmol) in CH_2Cl_2 (6 mL) was added to the reaction mixture. The reaction mixture was stirred overnight. Following this time, the reaction was filtered and concentrated under reduced pressure to provide the bisimine **8** which was analyzed by NMR to confirm the presence of the desired imine protons at δ 8.71 and 8.69 ppm.

Compound 14. To a stirred solution of compound **13** (2.4 g, 5.2 mmol) in CH_2Cl_2 (21 mL) at 0 °C was added a solution of $NaBH_4$ (1.2 g, 31.9 mmol) in MeOH (5.3 mL) via an additional funnel. The reaction mixture was allowed to slowly warm to room temperature with stirring overnight. The reaction mixture was quenched with 25 mL of saturated aqueous K_2CO_3. The aqueous layer and the organic layers were separated. The aqueous layer was extracted with CH_2Cl_2 (3 x 25 mL) and the combined organic layers were washed with saturated aqueous $NaHCO_3$, (2 x 25 mL), and brine (2 x 25 mL), dried over $MgSO_4$ and filtered. The filtrate was concentrated under reduced pressure to provide the crude product, compound **14,** as a pale yellow oil which was purified by flash chromatography on normal phase silica gel (40 gram column) using the following gradient program at 60 mL/min: 100% CH_2Cl_2 + 0.5% TEA for 3 column volumes, then ramp to 5% MeOH-CH_2Cl_2 + 0.5% TEA over 20 column volumes, finally holding at 5% MeOH-CH_2Cl_2 + 0.5% TEA for 5 column volumes. The column eluant was monitored at 285 nm and the fractions containing the purified material were pooled and concentrated under reduced pressure. The purified diamine compound **14** was obtained as a colorless oil that was further dried under high vacuum and analyzed by LCMS (ESI) 443 $[M+H]^+$.

Compound 15. To a solution of the diamine compound **14** (0.95 g, 2.15 mmol) in anhydrous CH_2Cl_2 (21.5 mL) was added imidazole (0.6 g, 8.62 mmol) and *tert*-butyldimethylsilyl chloride (0.66 g, 4.3 mmol). The reaction mixture was stirred for 16 h at room temperature and then quenched with saturated aqueous $NaHCO_3$ (25 mL). The aqueous and organic layers were separated and the aqueous layer was extracted with CH_2Cl_2 (3 x 25 mL). The combined organic layers were washed with saturated aqueous $NaHCO_3$, (2 x 25 mL), and brine, dried over $MgSO_4$ and filtered. The filtrate was concentrated under reduced pressure to provide the crude product **15** as a oil which was purified by flash chromatography on normal phase silica gel (40 gram column) using the following gradient program at 40 mL/min: 100% CH_2Cl_2 + 0.5% TEA for 2 column volumes, then ramp to 10% MeOH-CH_2Cl_2 + 0.5% triethylamine over 20 column volumes, finally holding at 10% MeOH-CH_2Cl_2 + 0.5% triethylamine for 4 column volumes. The column eluant was monitored at 285 nm and fractions containing the purified material were pooled and concentrated under reduced pressure to yield purified *tert*-butyldimethylsilyl ether **15** as a colorless oil (1.11 g, 2.0 mmol, 93%) which was further dried under high vacuum and then analyzed by LCMS (ESI) 558 $(M+H)^+$.

Compound 16. The diamine compound **15** (1.11 g, 1.99 mmol) was dissolved in a solution containing triethylphosphite (25 mL, 146 mmol) and $CHCl_3$ (10 mL). Paraformaldehyde (0.5 g) was added to the reaction mixture and the mixture was heated and maintained at a temperature of 35 °C for 4 days. At the end of the stipulated time, the reaction mixture was checked by LCMS, which indicated that the reaction had not proceeded to completion. An aliquot (1 mL) from the reaction mixture was added to a microwave reaction vessel followed by addition of paraformaldehyde 100 mg. The mixture was subjected to microwave irradiation for 10 min at 85 °C. Following the microwave irradiation, an additional portion of paraformaldehyde (100 mg) was added and the mixture was heated for 20 min at a temperature of 85 °C in the microwave. LCMS analysis of the reaction mixture, indicated further conversion to the product **16**. Heating the aliquot for an additional 60 minutes at 100°C under microwave irradiation resulted in complete conversion to product. The remainder of the reaction mixture was divided between 5 microwave tubes and each of the tubes was treated with paraformaldehyde (500 mg) and subjected to microwave heating at a temperature of 100 °C for 90 minutes to provide good conversion to the product **16**. The tubes were pooled and concentrated under reduced pressure. The residue was co-evaporated with three portions of ethanol and placed under high vacuum overnight. The crude product was purified by flash

chromatography on normal phase silica gel (120 gram column) using the following gradient program at 80 mL/min: 88% EtOAc-hexanes + 0.5% TEA for 20 column volumes. The column eluant was monitored at 277 nm and the fractions containing the purified material were pooled and concentrated under reduced pressure. The purified compound **16** was obtained as a colorless oil that was dried under high vacuum, and analyzed by LCMS (ESI) 857 [M+H]+, 879 [M+Na]+.

Compound 17. To a stirred solution of **16** (0.26 g, 0.30 mmol) in CH_2Cl_2 (3.0 mL) at room temperature was added bromotrimethylsilane (0.20 mL, 1.5 mmol). The reaction mixture was stirred at room temperature and the progress of the reaction was monitored by LCMS. After 18 hours the reaction was deemed to be complete, the major product being bisphosphonic acid **17** which was free of the bisphosphonate starting material **16**. The solvent was evaporated under reduced pressure and the residue further dried under high vacuum for 15 min to provide a colorless foam comprising the bisphosphonic acid **17** and lesser amounts of compounds in which acetonide(s) and/or silyl group were deprotected. This crude product mixture was used directly to prepare iron complex **Fe-5**.

Compound Fe-5. Crude reaction product containing **17** was dissolved in dioxane (1 mL); to this was added $FeCl_3$ hexahydrate (88 mg, 0.26 mmol) in water (1 mL) and then 4M HCl in dioxane (1 mL, 4 mmol). The reaction mixture was stirred at room temperature and progress of the reaction was monitored by LCMS. The reaction appeared to be complete after 2.5 hours. The reaction mixture was then quenched with excess saturated aqueous $NaHCO_3$ and diluted with CH_2Cl_2. The aqueous layer and the organic layers were separated. The aqueous layer (pH ~8) containing the product iron complex **Fe-5** was extracted with CH_2Cl_2 (2 x 20 mL) and was then filtered through a sintered glass funnel and further concentrated under reduced pressure to remove trace volatiles. Iron complex **Fe-5** was obtained as a deep red solution (approximately 30 mL) which was filtered through a 30000 MWCO filter and analyzed by LCMS (ESI) 602 (M-H)$^-$, λ_{max} (DI) = 466nm.

Imaging Data

Imaging data collected in this study is shown is shown in the attached spreadsheet (Imaging data Supplemental Material FeHBED analogs.xls). Each table row represents the data collected from a single animal, grouped by the agent that was administered. Empty cells represent values that were not collected for the specific animal, typically because an acceptable image slice was not acquired.

Signal enhancement (SE) for a tissue is calculated by dividing the MRI signal at 5 m post injection by the MRI signal pre-injection. SE for the kidney cortex is taken from the kidney cortex. The statistics to support statements in the manuscript are presented below

Naïve model

Kidney Cortex

Test for equal variances. Kidney cortex SE in the naive model for three CAs (**Fe-1**, **Fe-2** and **Gd-1**) may be assumed to have equal variances; the pooled standard deviation was used for ANOVA.

Tests

Method	Test Statistic	P-Value
Multiple comparisons	—	0.828
Levene	0.13	0.879

ANOVA . An ANOVA shows that the SE in the kidney cortex provided by **Gd-1** is greater than either **Fe-1** or **Fe-2** ($p < 0.05$).

Grouping via Tukey Method and 95% Confidence

Agent ID	N	Mean	Grouping	
Gd-1	6	1.44	A	
Fe-2	7	1.23		B
Fe-1	3	1.17		B

Tukey Simultaneous Tests for Differences of Means

Difference of Levels	Difference of Means	Adjusted P-Value
Fe-2 - Fe-1	0.07	0.74
Gd-1 - Fe-1	0.28	0.03
Gd-1 - Fe-2	0.21	0.04

Individual confidence level = 97.95%

Liver

Test for equal variances. Liver SE in the naive model for three CAs (**Fe-1**, **Fe-2** and **Gd-1**) may be assumed to have equal variances; the pooled standard deviation was used for ANOVA.

Tests

Method	Test Statistic	P-Value
Multiple comparisons	—	0.676
Levene	0.413	0.671

ANOVA. An ANOVA shows that the liver SE in the naïve model provided by **Fe-1** is greater than either **Gd-1** or **Fe-2**; the difference is highly significant, $p<0.0001$.

Grouping via Tukey Method and 95% Confidence

Agent ID	N	Mean	Grouping	
Fe-1	3	1.49	A	
Gd-1	6	1.06		B
Fe-2	7	1.02		B

Tukey Simultaneous Tests for Differences of Means

Difference of Levels	Difference of Means	Adjusted P-Value
Fe-2 - Fe-1	-0.47	0.00002
Gd-1 - Fe-1	-0.43	0.00005
Gd-1 - Fe-2	0.03	0.81116

Individual confidence level = 97.95%

Tumor model

Kidney cortex

Test for equal variances. Kidney cortex SE in the tumor model for five CAs (**Fe-2**, **Fe-3**, **Fe-4**, **Fe-5** and **Gd-1**) may be assumed to have equal variances; the pooled standard deviation was used for ANOVA.

Tests

Method	Test Statistic	P-Value
Multiple comparisons	—	0.827
Levene	0.13	0.752

ANOVA. An ANOVA shows that kidney cortex SE in the tumor model afforded by **Gd-1**, **Fe-3**, **Fe-4** and **Fe-5** are equivalent ($p > 0.18$). Kidney cortex SE from **Gd-1** and **Fe-3** is greater than **Fe-2** ($p \leq 0.5$).

Grouping via Tukey Method and 95% Confidence

Agent ID	N	Mean	Grouping	
Gd-1	8	1.51	A	
Fe-3	9	1.46	A	
Fe-4	4	1.34	A	B
Fe-5	5	1.29	A	B
Fe-2	4	1.16		B

Pooled Standard Deviation = 0.13

Tukey Simultaneous Tests for Differences of Means

Difference of Levels	Difference of Means	Adjusted P-Value
Fe-3 - Fe-2	0.29	0.007
Fe-4 - Fe-2	0.18	0.326
Fe-5 - Fe-2	0.13	0.587
Gd-1 - Fe-2	0.34	0.002
Fe-4 - Fe-3	-0.12	0.578
Fe-5 - Fe-3	-0.16	0.183
Gd-1 - Fe-3	0.05	0.923
Fe-5 - Fe-4	-0.05	0.979
Gd-1 - Fe-4	0.17	0.246
Gd-1 - Fe-5	0.22	0.050

Individual confidence level = 99.29%

Liver

Test for equal variances. Liver SE in the tumor model for five CAs (**Fe-2**, **Fe-3**, **Fe-4**, **Fe-5** and **Gd-1**) may be assumed to have equal variances; the pooled standard deviation was used for ANOVA.

Tests

Method	Test Statistic	P-Value
Multiple comparisons	—	0.641
Levene	0.92	0.467

ANOVA. An ANOVA shows that the liver SE in the tumor model provided by **Fe-3** is greater than **Fe-2** (p = 0.006), differences in SE between other pairs are not significant (p > 0.083).

Grouping via Tukey Method and 95% Confidence

Agent ID	N	Mean	Grouping	
Fe-3	10	1.30	A	
Gd-1	8	1.13	A	B
Fe-5	5	1.09	A	B
Fe-4	4	1.06	A	B
Fe-2	5	0.98		B

Pooled Standard Deviation = 0.15

Tukey Simultaneous Tests for Differences of Means

Difference of Levels	Difference of Means	Adjusted P-Value
Fe-3 - Fe-2	0.32	0.006
Fe-4 - Fe-2	0.08	0.940
Fe-5 - Fe-2	0.10	0.814
Gd-1 - Fe-2	0.15	0.425
Fe-4 - Fe-3	-0.24	0.083
Fe-5 - Fe-3	-0.21	0.103
Gd-1 - Fe-3	-0.17	0.169
Fe-5 - Fe-4	0.03	0.999
Gd-1 - Fe-4	0.07	0.931
Gd-1 - Fe-5	0.05	0.982

Individual confidence level = 99.30%

Tumor

Test for equal variances. Tumor SE in the tumor model for five CAs (**Fe-2**, **Fe-3**, **Fe-4**, **Fe-5** and **Gd-1**) may be assumed to have equal variances; the pooled standard deviation was used for ANOVA.

Tests

Method	Test Statistic	P-Value
Multiple comparisons	—	0.442
Levene	1.05	0.399

ANOVA. An ANOVA shows that the SE in the tumor afforded by **Gd-1** is greater than all the iron agents evaluated in the tumor model (p < 0.0005). The iron agents were equivalent to each other (p > 0.06).

Grouping via Tukey Method and 95% Confidence

Agent ID	N	Mean	Grouping	
Gd-1	8	1.82	A	
Fe-3	10	1.41		B
Fe-5	5	1.29		B
Fe-4	6	1.26		B
Fe-2	5	1.15		B

Pooled Standard Deviation = 0.17

Tukey Simultaneous Tests for Differences of Means

Difference of Levels	Difference of Means	Adjusted P-Value
Fe-3 - Fe-2	0.26	0.0637
Fe-4 - Fe-2	0.12	0.7972
Fe-5 - Fe-2	0.14	0.6830
Gd-1 - Fe-2	0.68	0.0000
Fe-4 - Fe-3	-0.15	0.4740
Fe-5 - Fe-3	-0.12	0.7075
Gd-1 - Fe-3	0.41	0.0002
Fe-5 - Fe-4	0.03	0.9989
Gd-1 - Fe-4	0.56	0.0000
Gd-1 - Fe-5	0.53	0.0001

Individual confidence level = 99.31%

References

1. Fulmer GR, Miller AJM, Sherden NH, Gottlieb HE, Nudelman A, Stoltz BM, et al. NMR Chemical Shifts of Trace Impurities: Common Laboratory Solvents, Organics, and Gases in Deuterated Solvents Relevant to the Organometallic Chemist. Organometallics. 2010 May 10;29(9):2176–9.

2. Mathias CJ, Sun YZ, Welch MJ, Green MA, Thomas JA, Wade KR, et al. Targeting radiopharmaceuticals: comparative biodistribution studies of gallium and indium complexes of multidentate ligands. Int J Rad Appl Instrum B. 1988;15(1):69–81.
